# Aboriginal families living with MJD in remote Australia: questions of access and equity

**DOI:** 10.1186/s12939-024-02228-x

**Published:** 2024-09-18

**Authors:** Libby Massey, John Gilroy, Emma Kowal, Denise Doolan, Alan Clough

**Affiliations:** 1 Division of Tropical Health and Medicine, James Cook University, Cairns, QLD Australia; 2 MJD Foundation, Alyangula, NT Australia; 3 Australian Institute of Tropical Health and Medicine, James Cook University, Cairns, QLD Australia; 4 The Charles Perkins Centre, The University of Sydney, Sydney, Australia; 5 Alfred Deakin Institute, Deakin University, Melbourne, Australia

**Keywords:** Equity, Medically assisted reproduction, Access, Barriers, Remote Australia, Machado-Joseph disease

## Abstract

Managing genetic disease using medically assisted reproductive technology is increasingly promoted as a feasible option, given revolutionary advances in genomics. Far less attention has been directed to the issue of whether there is equitable access to this option. Context and circumstance determine equitable access; however, reporting has drawn overwhelmingly from affluent Anglo-western populations in developed countries. The experiences of poorer, less educated subpopulations within affluent countries and populations in less developed countries are underreported. The ability of consumers to understand the opportunities and risks of medically assisted reproductive technology is likewise not well described in the literature despite significant technological complexity and evidence that genetic disease may be overrepresented within some disadvantaged population groups.

Equity is achieved by identifying barriers and allocating appropriate resources to enable understanding and access. In the case of utilising medically assisted technology, social and power relationships, regulations, and the presumptions of authority figures and policymakers reduce equitable access. Physical or cultural marginalisation from mainstream health services may result in reduced access to genetic and prenatal testing, in-vitro fertilisation and genetic screening of embryos necessary for medically assisted reproduction. Cost and regulatory frameworks can likewise limit opportunities to engage with services. Moreover, the quality of the information provided to prospective users of the technology and how it is received governs understanding of prevention and inhibits adequately informed choice.

Best practice care and adequately informed choice can only be achieved by conscientiously attending to these accessibility issues. Deep engagement with at-risk people and critical reflection on mainstream accepted standpoints is required. This paper outlines issues associated with engaging with medically assisted reproduction encountered by Aboriginal families living with Machado-Joseph Disease in some of the most remote areas of Australia. It is the right of these families to access such technologies regardless of where they live. Current barriers to access raise important questions for service providers with implications for practice as new technologies increasingly become part of standard medical care.

## Background

### Genetic disease management has been approached from an Anglo-Western perspective and utilisation is variable and affected by context

Genomics and the treatment of genetic disease are rapidly emerging fields globally [1]. However, despite rapid progress in identifying pathogenic variants, many genetic conditions remain stubbornly incurable. Without treatment options, disease mitigation often requires those who carry pathogenic variants to align their decision-making with biomedical principles of ‘genetic responsibility’, which involves a commitment to not passing the affected genes to their children [2]. Strategies currently recommended for managing genetic disease require high levels of health literacy and include; genetic education, genetic screening or testing, and either termination of pregnancy, or using medically assisted reproductive technology (MAR) such as IVF with preimplantation genetic testing (PGT) and PGT for monogenetic diseases (PGT-M) [3].

How affected couples engage with these strategies, the range of influences and their decision-making processes are not comprehensively described [4]. The principles that underpin genetic disease management are grounded in scientific knowledge and pragmatism, and the dominant discourse draws from Anglo–western published literature from wealthy industrialised countries [5]. This literature reports a wide range of uptake and understanding of the recommended management strategies and reveals that many elements contribute to the perspectives of people who live with genetic disease. The nature of the condition, socioeconomic factors, culture, religion and access to technology are highly influential [6–10].

Practical access is mediated by a wide range of regulatory and legal environments for those who want to engage with genomic technology, even within industrialised countries. Elements of MAR, such as preimplantation genetic testing (PGT), are prohibited in some jurisdictions, such as Italy and Ireland, or highly regulated and permissible only under specific conditions, for example in France [11]. Other countries, such as the USA and Brazil, impose very little regulation; however, the procedures have prohibitive costs, and no financial assistance is available, effectively restricting use to those with financial means. In Australia, following sustained lobbying arguing beneficence, procreative autonomy and justice [12], the costs of PGT for couples with a high risk of genetic disease were subsidised through the national medical insurance scheme in late 2021.

Broader social and economic factors also contribute to access. Carrier screening for disabling and fatal recessive conditions is increasingly available, acceptable, and even regarded as normative in some jurisdictions, such as in Israel [10], or where there are high rates of recessive conditions within the Ashkenazi Jewish and Arab populations [13].Here, demand is consistent with socialised acceptance of screening and MAR techniques, endorsement by the medical profession, financial assistance and ready access through government programs [10].

Across jurisdictions, higher income and education correspond to better genetic literacy and engagement with genetic services [14]. Traditional or conservative cultural, religious and moral perspectives are generally associated with lower engagement with genetic services [9]. Access to genomic intervention is, therefore, situational and highly variable and achieving equity will require attention to specific issues experienced by subgroups within populations. In the case of Australia, Aboriginal people experiencing genetic disease are confronted with complex systemic and ingrained barriers that will require thoughtful collaborative redress.

### Marginalised communities are not just in third-world countries. In Australia Aboriginal people living in remote communities are marginalised.

First Nations people are over-represented in statistics that indicate marginalisation from health care systems and resources worldwide [3]. Despite high standards of care, and a publicly funded system, Australian reports indicate that clinical care of Aboriginal people is unintentionally impacted by institutionalised racism, and implicit systemic bias [15, 16]. Diagnostic investigations, procedures, care planning, treatments and adherence to best practice treatment guidelines are all affected [3]. Unsurprisingly, Aboriginal people engage less with health care services and experience poorer health across their lifespan [17].

Additional barriers are present for Aboriginal people who live in remote locations. These communities experience harsh weather conditions and have very limited technical and environmental infrastructure. The large family groups who live in the communities often maintain strong, traditional cultural values, including adherence to moiety-based kinship systems and avoidance relationships [18]. Most people speak English as a second or subsequent language, and a high value is placed on continuity and trust between health service providers and clients [19, 20].

A poorly coordinated combination of government and Aboriginal community-controlled health services (ACCHS) provides primary health care to these isolated outposts. These separate systems have limited capacity to share records between them, or with tertiary referral centres, despite the high and seasonal mobility of the local Aboriginal people. Non-Aboriginal health professionals employed on short-term contracts provide most community-based health care [19, 20]. Access to regional hospitals for specialist care involves substantial travel, cost, dislocation from support systems, and health care delivery grounded in Anglo-western perspectives and the English language. In the case of genetic services to the Northern Territory, there is no resident medical specialist, and care is provided on a fly-in fly-out basis from a southern capital city [3, 20]. All these factors are barriers to effective engagement with health care and the comfort and well-being of the patient [3, 20, 21].

### MJD in Aboriginal people living in remote communities in northern Australia

Machado-Joseph disease (MJD), also known as spinocerebellar ataxia type-3, is a lethal, genetic, progressive neurodegenerative ataxia caused by a pathogenic CAG expansion on chromosome 14q [22]. The genetic change causes an abnormal protein called ataxin-3 to be produced, which degrades muscle and nerve function leading to devastating long-term disability. Functional impacts include progressive deterioration of balance, coordination, continence and vision [23, 24]. Non-motor systems are also impacted, resulting in severely disturbed sleep and mildly impaired executive function [25].

Genetic inheritance of MJD follows an autosomal dominant pattern. Therefore, there is a fifty per cent risk to each child conceived by an affected parent. It is also unstable in transmission from parent to child, resulting in earlier onset and progression to severe symptoms between generations [23]. Consequently, there may be several generations of family members experiencing a range of disease severity simultaneously. Symptoms progress slowly over 20 years and ultimately render affected people unable to move, communicate, swallow or toilet independently. There are no effective treatments available for MJD [26].

**Fig. 1:**
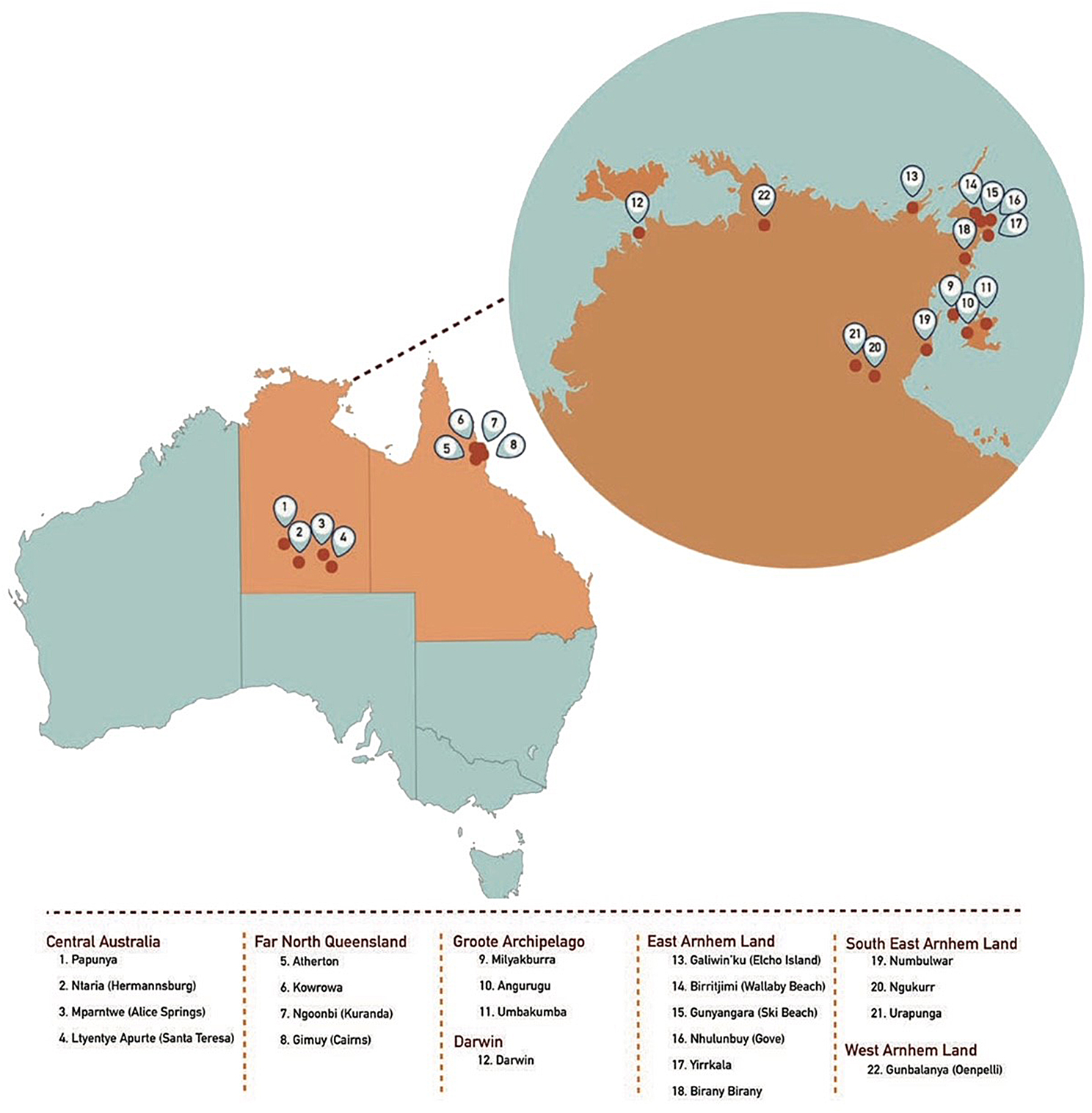
Australian MJD Locations

Worldwide the prevalence of MJD ranges between 1 and 5 per 100 000 [27]. It is a rare disease but the most frequent autosomal dominant ataxia and is experienced in diverse populations worldwide, including Brazil, China, Japan, Portugal and the Netherlands [28]. The disease has an extensive history in remote north Australian Aboriginal communities and has been clinically documented from the 1960s [29, 30]. Local prevalence rates in these communities are thought to be among the highest known anywhere (Groote Eylandt Archipelago (~ 743/100,000: Azores Archipelago ~ 39/100,000) [24, 31]. Although how the disease entered into the population is uncertain, families in this region have a haplotype correlated with an Asian origin and likely related to longstanding trading relationships with Asian neighbours (Fig. 1) [32].

### What is known about attitudes of Australian Aboriginal people with MJD towards engaging with genomics?

Australian Aboriginal families experiencing MJD in remote communities are interested in exploring information about the disease and its potential management. Demonstrating this, some have contributed to recent publications outlining local and systemic barriers to care and providing evidence of culturally safe practices in genetic education and counselling [19, 20, 33]. In several Arnhem Land communities, five generations have been clinically affected, and the progressive lowering age of onset and rapid progression in recent generations is a growing concern. With the support of their local land council, senior women from affected families on Groote Eylandt have initiated a research project to investigate how medically assisted reproductive technology principles, including prenatal genetic diagnosis and preimplantation genetic diagnosis, align with their worldview [34].

While this research is still ongoing, it demonstrates an openness to engaging with genetic and screening information that has been previously reported with foetal anomaly screening in similar remote Aboriginal communities [35]. Unfortunately, despite this openness, residents’ opportunities to engage with MAR are likely to encounter many of the same barriers at the individual, interpersonal and health system levels discussed above [3, 20]. To fully engage with the suite of medically assisted reproductive options, at-risk family members need to know their genetic status, entailing genetic counselling and pre-symptomatic or diagnostic genetic testing. Engagement with genetic services to achieve this is currently low, with referral, scheduling and attendance rates for Aboriginal people below population parity, and especially poor for people from remote areas [3]. Factors that would improve engagement include attention to cultural safety, including gender-specific services, assistance with logistics to enable appointments and communication appropriate for people for whom English is not first language and who have limited literacy.

Other subtler barriers are potentially contentious. Aboriginal families living in remote communities may prefer genetic care that aligns with notions of collective, rather than individual ownership of information and responsibility. Important traditional relationship structures frequently require consent and attendance at appointments by people beyond the individual concerned [19]. Respect for privacy and individual autonomy is ingrained in the Australian healthcare sector, and attempts to change current practices may be challenged by health professionals in the mistaken belief that collective or stratified management of information and alternative prioritisation of issues are detrimental to patient care. On the contrary, adopting culturally sensitive practices in the provision of genetic care and testing will rebalance the health professional-patient relationship and allow for more effective clinical interactions.

Along with these systemic issues, there is also evidence of inconsistent actions of health care providers who, when faced with relaying complex information to people who do not speak English, and a lack of readily available learning materials, sometimes resort to actions consistent with a subjective perception of the Aboriginal persons’ interest and literacy such as assumptions of the acceptability of the risks in prenatal testing [5, 35].

### What can be done?

Aboriginal people living in remote regions have rights to equitable access to genetic care enshrined in the United Nations Declaration on the Rights of Indigenous People [36]. The Australian Government recognises this within its *National Health Genomics Policy Framework* [37]. The framework has five strategic priorities and has identified engagement with community as a priority action area. It outlines the need to identify barriers to equity of access and minimise them, specifying the impact of location, cost, availability, and cultural acceptability of services. Improving access and engagement with these services requires listening to the advice of those who will use the services (Table 1).

**Table 1:** National health genomics policy key priorities

Person-centred approach	Delivering high-quality care for people through a person-centred approach to genomics.
Workforce	Building a skilled workforce that is literate in genomics.
Financing	Ensuring sustainable and strategic investment in cost-effective genomics.
Services	Maximising quality, safety and clinical utility of genomics in health care.
Data	Responsible collection, storage, use and management of genomic data.

**Table:** 

**Priority Area for Action 1.5**	
Identify barriers to equity of access and develop a national approach to address these, noting that access is multi-dimensional and includes location, cost, availability and appropriateness (including cultural acceptability). This includes, but is not limited to:	
• exploring barriers to the uptake of genomic services including the potential for discrimination (life insurance, employment, lifestyle, access to services); and.	
• evaluating the delivery of genomic services in terms of being accessible, appropriate and culturally secure and responsive for Aboriginal and Torres Strait Islander peoples.	

There is emerging evidence that culturally tailored services improve outcomes in genetic care [38–40]. To provide equitable access to genetic care for Aboriginal people living in remote Northern Territory communities, it will be necessary for the relevant health care provider, in this case The *Northern Territory Department of Health* to pay close attention to aligning genetic services with cultural safety. This can be achieved by implementing processes which incorporate improved engagement with Aboriginal communities, for example by establishing place based clinics, prioritising genetic knowledge within Aboriginal Health Worker training and forming strategic collaborations with relevant stakeholders such as local Non-Government Organisations (NGO) and Aboriginal Controlled Community Health Organisations (ACCHO) in communities where genetic disease rates are high. These strategies will enable two-way learning, honouring and elevating the cultural knowledge of Aboriginal people and provide avenues for cultural safety training for non-Aboriginal genetic health practitioners. Attention should additionally be paid to developing appropriate resources and educational opportunities to develop higher levels of genetic health literacy within at risk cohorts to facilitate adequately informed decision making [19, 33]. These strategies will enable the co-design of services with Aboriginal people to ensure better care at each stage of the patient journey and improve the linkage of systems. In practical terms, ensuring that Aboriginal people have the opportunity to access first language and cultural support for all engagement with non- Aboriginal genetic care providers is vital and will assist in ameliorating implicit bias [3, 5]. Ongoing research and quality improvement strategies to monitor the efficacy and uptake of services will also be critical. People living with MJD have already contributed knowledge by recommending client-led services, respecting traditional orientation to familial disease causes, focusing on sustained trusting relationships and incorporating whole-of-family practice [19, 20, 33]. Best practice care can only be achieved by conscientiously attending to these issues. Neglecting these issues will leave Aboriginal people living with MJD without access to this vital aspect of care.

Significant physical, social and emotional costs are already being born by individuals and families with MJD in remote Australia and this is destined to worsen as more people experience symptoms, and their loved ones become carers. The tools and understanding to engage with technology and make informed choices for their future and that of their children are currently inaccessible. It is incumbent on those who design and deliver these services to evaluate the healthcare and personal costs of retaining current practices which demonstrate limited engagement and accessibility and move toward a more flexible, integrated and client focussed approach.

## Data Availability

All information in the paper are available according to the terms of the publisher.
